# Association of diabetes mellitus with clinical outcomes in patients with different coronary artery stenosis

**DOI:** 10.1186/s12933-021-01403-6

**Published:** 2021-10-23

**Authors:** Hui-Wen Zhang, Jing-Lu Jin, Ye-Xuan Cao, Yuan-Lin Guo, Na-Qiong Wu, Cheng-Gang Zhu, Rui-Xia Xu, Qian Dong, Jian-Jun Li

**Affiliations:** State Key Laboratory of Cardiovascular Disease, National Clinical Research Center for Cardiovascular Diseases, Fu Wai Hospital, National Center for Cardiovascular Diseases, Chinese Academy of Medical Sciences and Peking Union Medical College, No 167 BeiLiShi Road, XiCheng District, Beijing, 100037 China

**Keywords:** Diabetes mellitus, Coronary artery stenosis, Cardiovascular events

## Abstract

**Background:**

It has been demonstrated that patients with type 2 diabetes mellitus (DM) is associated with increased cardiovascular risk. However, little is known regarding the long-term prognosis in diabetic patients who experience mild-to-intermediate coronary artery stenosis (CAS). This study was to assess the clinical outcomes of diabetic patients with different severity of CAS.

**Methods:**

We consecutively enrolled 10,940 patients hospitalized due to angina-like chest pain and followed up for major adverse cardiovascular events (MACEs) covering cardiac death, myocardial infarction, ischemic stroke, unplanned coronary revascularization and angina-related hospitalization. According to coronary angiography, patients were divided into non-obstructive CAS (NOCAS, < 50% stenosis), intermediate CAS (ICAS, 50–69% stenosis), and severe CAS (SCAS, 70–100% stenosis) subgroups, and were further categorized into six groups as NOCAS with DM and non-DM, ICAS with DM and non-DM, and SCAS with DM and non-DM.

**Results:**

During a median follow-up of 40 months, 1,017 (11.1%) MACEs occurred. In patients with ICAS or SCAS, the incidence of events was higher when patients coexisted with DM (p < 0.05, respectively). In subgroup analyses, patients with ICAS and DM, SCAS and non-DM, SCAS and DM had increased risk of events [adjusted hazard ratio (HR): 1.709, 95% confidence interval (CI) 1.106–2.641, p = 0.016; HR: 1.911, 95% CI 1.460–2.501, p < 0.001; HR: 2.053, 95% CI 1.514–2.782, p < 0.001] compared to ones with NOCAS and non-DM. Besides, the Kaplan–Meier curves indicated the highest risk of MACEs in patients with SCAS and DM than others (p < 0.001).

**Conclusions:**

Diabetic patients with ICAS had the worse outcome, which was comparable to patients with SCAS alone.

## Introduction

Type 2 diabetes mellitus (DM) is a major risk factor of cardiovascular disease [1–3]. The cardiovascular mortality is increased by more than two-fold in diabetic patients compared to those without [2–5]. Previous studies have revealed that patients with DM are also subjected to worse cardiovascular outcomes [2–8], especially in those coexisted with coronary artery diseases (CAD) [7, 8]. Notably, in diabetic patients with CAD, the pathological feature of coronary lesions is more complicated, which is characterized by calcified, diffuse, multivessel disease, and greater atherosclerotic plaque burden [9, 10]. Therefore, these patients often require assessment of coronary lesions and revascularization in addition to optimal medical therapy to control angina [11].

Clinically, a quite large proportion of patients (30% to 70%) receiving coronary angiography (CAG) due to angina-like chest pain do not present severe obstructive stenosis in coronary arteries [12–14]. Importantly, most of these patients presented a mild or moderate coronary stenosis but continue to experience recurrent angina. Additionally, they might be also subjected to high healthcare costs due to repeating hospitalization and coronary artery angiograms, and even high risk of adverse cardiovascular event. [14–16]. More recently, an expert consensus document published in European Heart Journal stressed on this issue, indicating that patients with ischemia with non-obstructive coronary arteries were also independently associated with poor clinical outcomes and required future ongoing research for comprehensive management in such patients [17].

To our knowledge, less data is currently available regarding the joint impact of DM on clinical outcomes in a large cohort who suffer from mild-to-intermediate coronary artery stenosis (CAS). Therefore, the aim of the present study was to explore the prognostic association of type 2 diabetic patients with different CAS assessed by selective CAG in a large Chinese cohort.

## Material and methods

### Study population

**Fig. 1:**
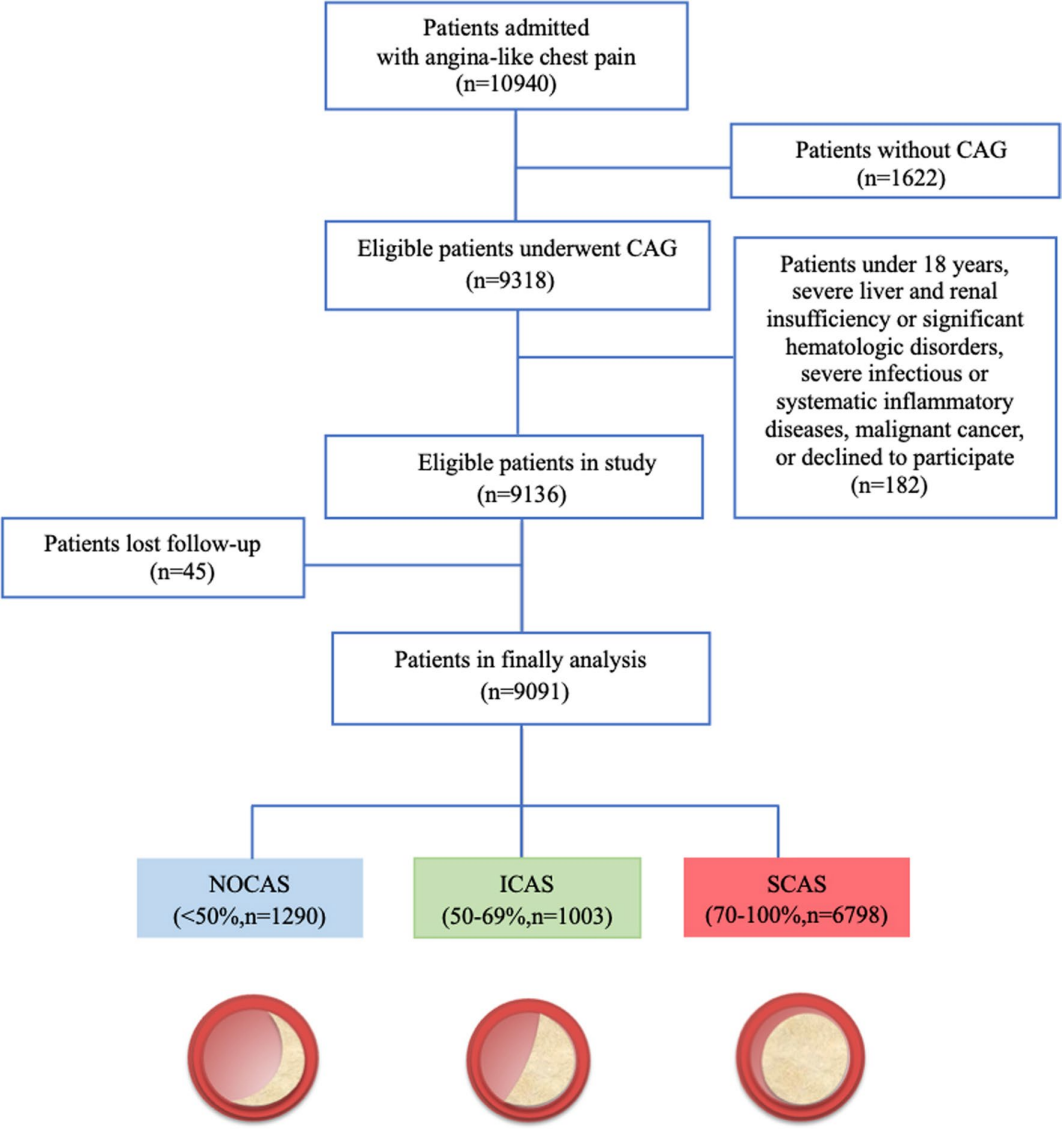
Study flowchart. (CAG: coronary angiography; NOCAS: non-obstructive coronary artery stenosis; ICAS: intermediate coronary artery stenosis; SCAS: severe coronary artery stenosis)

From April 2011 to March 2018, a total of 10,940 consecutive patients were hospitalized due to angina-like chest pain in FuWai Hospital (National Center for Cardiovascular Diseases, Beijing, China). The study flowchart of this observational prospective study was shown in Fig. 1. Briefly, patients who met the exclusion criteria: under 18 years-old, severe liver and renal dysfunction, severe infectious or systematic inflammatory diseases, significant hematologic disorders, malignant cancer, decline to participate; patients without CAG; or patients lost follow-up were excluded. The indications for CAG including patients with angina‐like chest pain, and/or with positive treadmill exercise test, and/or with significant coronary stenosis assessed by computed tomography angiography (CTA) [18]. Finally, a total of 9091 eligible patients received CAG were enrolled, including 6918 stable angina, 1380 unstable angina, and 793 old myocardial infarction (MI). Among them, 4493 patients received coronary revascularization in hospital, including 4306 patients underwent percutaneous coronary intervention (PCI), and 187 patients did coronary artery bypass graft (CABG).

This study complied with the Declaration of Helsinki and was approved by the hospital ethical review board (Approval No.: 2013-442. FuWai Hospital and National Center for Cardiovascular Diseases, Beijing, China). The informed written consent was obtained from all patients.

### Definitions

The CAG was invasively obtained via heart catheterization using standard clinical protocols, and all coronary angiograms were reviewed by the experienced invasive cardiologists. According to result of CAG, patients were divided into non-obstructive CAS (NOCAS, < 50% luminal stenosis) group, intermediate CAS (ICAS, 50–69% luminal stenosis) group, and severe CAS (SCAS, 70–100% luminal stenosis) group [16, 19, 20]. DM was diagnosed according to the American Diabetes Association criteria [21], defined as the fasting plasma glucose ≥ 7.0 mmol/L (126 mg/dL), or 2-h plasma glucose of the oral glucose tolerance test ≥ 11.1 mmol/L (200 mg/dL), or hemoglobin A1c (HbA1c) level ≥ 6.5%, or patients with hypoglycemic drugs treatment. Diagnosed hypertension was defined with self-reported hypertension, and currently treatment of anti-hypertensive drugs. Undiagnosed hypertension was diagnosed with hospital recorded systolic blood pressure ≥ 140 mmHg and/or diastolic blood pressure ≥ 90 mmHg for three or more consecutive times using standard mercury sphygmomanometer by trained physicians according to the guideline [22]. Body mass index (BMI) was calculated by dividing weight in kilograms by height in square meters. The diagnosis of other diseases, family history of CAD, and treatments were collected as previous study reported [23].

The endpoints were defined as major adverse cardiovascular events (MACE) covering cardiac death, non-fatal MI or ischemic stroke, unplanned coronary revascularization (including PCI and/or CABG), and unstable angina-related hospitalization. The detailed methods of follow-up visit have been reported as our previous study [23]. Each subject was actively followed up at 6-month intervals after the initial hospitalization, with outpatient clinic interviews or telephone contact by our well-trained cardiologists or nurses, who were blinded to the objectives of the present study.

### Laboratory tests

Laboratory data were obtained from each patient with their venous blood samples taken after 12 h overnight at the initial entry in hospital, and all samples were stored in a − 80 °C refrigerator until test. The lipid profiles were measured by automatic biochemistry analyzer (Hitachi 7150, Tokyo, Japan) followed the methods of our previous study [23]. Detailly, the low-density lipoprotein cholesterol (LDL-C) concentrations were measured by selective solublilization method (low-density lipid cholesterol test kit; Kyowa Medex, Tokyo). High-density lipoprotein cholesterol (HDL-C) concentrations were analyzed using a homogeneous method (Determiner L HDL; Kyowa Medex, Tokyo). Total cholesterol, Triglyceride, Apolipoprotein A1 (ApoA1), and ApoB were measured by automatic biochemistry analyzer (Hitachi 7150, Tokyo, Japan) in an enzymatic assay. The fast glucose concentrations were measured by enzymatic hexokinase method. HbA1c was measured using Tosoh Automated Glycohemoglobin Analyser (HLC-723G8, Tokyo, Japan).

### Statistics

Continuous data were showed as mean with standard deviation, or median with interquartile ranges. The Student t-test, Mann–Whitney U test, or the Kruskal–Wallis test was performed for subgroup comparisons as appropriate. Categorical data was presented with percentage (%) and compared by Chi-square test, or Fisher’s exact test. The risks of event in different subgroups were compared by Kaplan–Meier survival curves and log-rank test. The associations among DM, coronary stenosis, and clinical outcomes were analyzed by the unadjusted and adjusted Cox regression analyses [showed with hazard ratios (HR), and 95% confidence intervals (CI)]. We performed statistical analyses with SPSS software version 23.0 (SPSS, Inc., Chicago, Illinois). A p value < 0.05 was considered statistical significance.

## Results

### Baseline characteristics

The baseline characteristics of enrolled patients were shown in Table 1. Among the subjects, the average age was 57.2 years old and 70.7% (n = 6431) were male. According to the result of CAG, patients were classified into three subgroups: NOCAS group (n = 1290), ICAS group (n = 1003), and SCAS group (n = 6798). Overall, 34.9% (n = 3173) of them had DM. Patients with more severe coronary stenosis showed higher prevalence of DM (SCAS vs ICAS vs NOCAS: 38.1% vs 29.6% vs 22.3%, p < 0.001), and higher levels of fast glucose (SCAS vs ICAS vs NOCAS: 5.95 ± 2.83 mmol/L vs 5.71 ± 1.57 mmol/L vs 5.47 ± 1.36 mmol/L, p < 0.001) and HbA1c (SCAS vs ICAS vs NOCAS: 6.37 ± 1.15% vs 6.16 ± 0.92% vs 6.00 ± 0.86%, p < 0.001). Besides, patients were more likely to have hypertension, dyslipidemia, current smoking, and family history of CAD with the increase in the degree of CAS (all p < 0.05).

**Table 1:** Baseline characteristics according to the severity of coronary artery stenosis

Variables	Total	NOCAS (< 50%)	ICAS (50–69%)	SCAS (70–100%)	P value for trend
Baseline characteristics					
Patients (n, %)	9091	1290 (14.2%)	1003 (11.0%)	6798 (74.8%)	
Age (years)	57.22 ± 10.37	55.36 ± 10.02	57.43 ± 9.85	57.54 ± 10.48	** < 0.001**
Male (n, %)	6431 (70.7%)	734 (56.9%)	644 (64.2%)	5053 (74.3%)	** < 0.001**
Hypertension (n, %)	5591 (61.5%)	680 (52.7%)	598 (59.6%)	4313 (63.4%)	** < 0.001**
Dyslipidemia (n, %)	6759 (74.3%)	860 (66.7%)	749 (74.7%)	5150 (75.8%)	** < 0.001**
Diabetes mellitus (n, %)	3173 (34.9%)	288 (22.3%)	297 (29.6%)	2588 (38.1%)	** < 0.001**
BMI (kg/m^2^)	25.89 ± 3.23	25.68 ± 3.41	25.81 ± 3.28	25.94 ± 3.19	**0.027**
Current smoking (n, %)	2880 (31.7%)	306 (23.7%)	288 (28.7%)	2286 (33.6%)	** < 0.001**
Family history of CAD (n, %)	1207 (13.3%)	145 (11.2%)	125 (12.5%)	937 (13.8%)	**0.034**
Laboratory data					
TC (mmol/L)	4.14 ± 1.12	4.22 ± 0.95	4.14 ± 1.06	4.13 ± 1.16	**0.012**
TG (mmol/L)	1.79 ± 1.23	1.77 ± 1.27	1.74 ± 1.19	1.80 ± 1.23	0.328
LDL-C (mmol/L)	2.51 ± 0.99	2.54 ± 0.83	2.52 ± 1.18	2.51 ± 0.92	0.428
HDL-C (mmol/L)	1.07 ± 0.29	1.12 ± 0.32	1.11 ± 0.29	1.05 ± 0.29	** < 0.001**
Apo A1 (g/L)	1.34 ± 0.30	1.41 ± 0.30	1.38 ± 0.29	1.32 ± 0.29	** < 0.001**
Apo B (g/L)	0.91 ± 0.28	0.92 ± 0.27	0.90 ± 0.28	0.92 ± 0.29	0.384
Glucose (mmol/L)	5.85 ± 2.56	5.47 ± 1.36	5.71 ± 1.57	5.95 ± 2.83	** < 0.001**
HbA1c (%)	6.29 ± 1.09	6.00 ± 0.86	6.16 ± 0.92	6.37 ± 1.15	** < 0.001**
Creatinine (mmol/L)	77.33 ± 17.46	73.23 ± 15.14	75.63 ± 19.53	78.36 ± 17.4	** < 0.001**
Treatment in hospital					
Aspirin (n, %)	8595 (94.5%)	1166 (90.4%)	959 (95.6%)	6470 (95.2%)	** < 0.001**
β-Blockers (n, %)	6846 (75.3%)	795 (61.6%)	700 (69.8%)	5351 (78.7%)	** < 0.001**
CCBs (n, %)	3356 (36.9%)	469 (36.4%)	399 (39.8%)	2488 (36.6%)	0.135
ACE inhibitors (n, %)	2045 (22.5%)	228 (17.7%)	174 (17.3%)	1643 (24.2%)	** < 0.001**
ARB (n, %)	2150 (23.6%)	265 (20.5%)	243 (24.2%)	1642 (24.2%)	**0.018**
Lipid-lowering medication (n, %)	8159 (89.7%)	1056 (81.9%)	898 (89.5%)	6205 (91.3%)	** < 0.001**
Anti-diabetes drugs (n, %)	3173 (34.9%)	288 (22.3%)	297 (29.6%)	2588 (38.1%)	** < 0.001**
MACE (n, %)	1017 (11.1%)	90 (7.0%)	94 (9.4%)	833 (12.3%)	** < 0.001**

*NOCAS* non-obstructive coronary artery stenosis, *ICAS* intermediate coronary artery stenosis, *SCAS* severe coronary artery stenosis, *BMI* body mass index, *LVEF* left ventricular ejection fraction, *CAD* coronary artery disease, *TC* total cholesterol, *TG* triglyceride, *LDL-C* low-density lipoprotein cholesterol, *HDL-C* high-density lipoprotein cholesterol, *Apo* apolipoprotein, *HbA1c* hemoglobin A1c, *CCBs* calcium channel blockers, *ACE* angiotensin converting enzyme, *ARB* angiotensin receptor blocker, *MACE* major adverse cardiovascular event

Values are expressed as the mean ± SD, or n (%). Bold P values are statistically significant

During a median follow-up of 40 months, there were 1,017 (11.1%) MACEs occurred, including 104 cardiac death, 75 non-fatal MI, 170 non-fatal stroke, 289 coronary revascularization, and 379 angina-related hospitalization. Notably, the incidence of MACEs increased in patients with SCAS when compared with those in ICAS and NOCAS groups (12.3% vs 9.4% vs 7.0%, p < 0.001).

### Diabetes, coronary stenosis, and outcomes

**Fig. 2:**
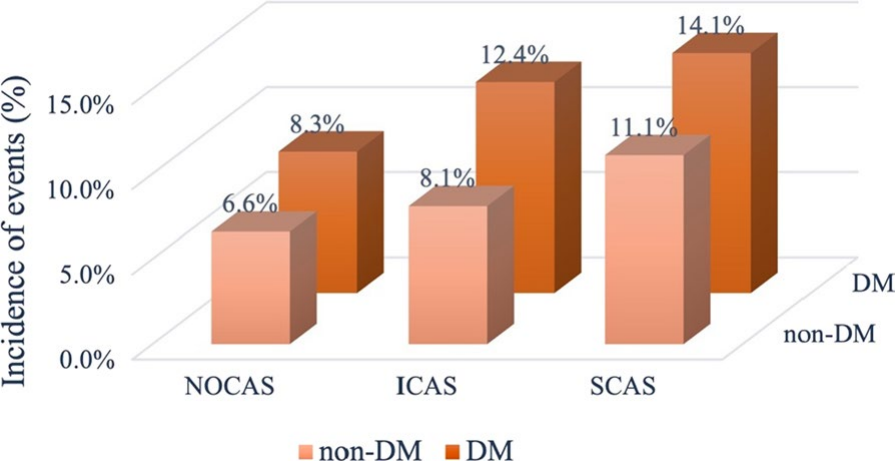
The incidence of cardiovascular events in different subgroups. (NOCAS: non-obstructive coronary artery stenosis; ICAS: intermediate coronary artery stenosis; SCAS: severe coronary artery stenosis; DM: diabetes mellitus)

As shown in Table 2, patients with MACEs had higher incidence of DM and hypertension (p < 0.001, respectively). They also showed increased levels of HbA1c (6.47 ± 1.20% vs 6.27 ± 1.09%, p < 0.001). Moreover, there was a higher percentage of SCAS in patients with MACEs compared to those without (81.9% vs 73.9%, p < 0.001). There was no significantly difference in coronary revascularization between patients with and without MACEs (p > 0.05). According to the status of glucose metabolism, the patients were further categorized into six subgroups as NOCAS with DM and non-DM group, ICAS with DM and non-DM group, and SCAS with DM and non-DM group. The incidence of MACEs in different subgroups were presented in Fig. 2. In patients with ICAS or SCAS, the incidence of MACEs was higher when patients coexisted with DM (ICAS: 12.4% vs 8.1%; SCAS: 14.1% vs 11.1%; p < 0.05, respectively). Patients in SCAS with DM group showed the highest incidence of MACEs (14.1%). Interestingly, among the patients with DM, the incidence of MACEs in patients with NOCAS was similar to that in ICAS without DM (8.3% vs 8.1%), and the incidence of MACEs in patients with ICAS was similar to that in SCAS without DM (12.4% vs 11.1%).

**Table 2:** Baseline characteristics in patients with or without cardiovascular events

Variables	Total	MACE	Non-MACE	P value
Baseline characteristics				
Patients (n, %)	9091	1017 (11.1%)	8074 (88.9%)	
Age (years)	57.22 ± 10.37	59.81 ± 9.98	56.89 ± 10.38	** < 0.001**
Male (n, %)	6431 (70.7%)	687 (67.6%)	5744 (71.1%)	**0.018**
Hypertension (n, %)	5591 (61.5%)	677 (66.6%)	4914 (60.9%)	** < 0.001**
Dyslipidemia (n, %)	6759 (74.3%)	751 (73.8%)	6008 (74.4%)	0.696
Diabetes mellitus (n, %)	3173 (34.9%)	425 (41.8%)	2748 (34.0%)	** < 0.001**
BMI (kg/m^2^)	25.89 ± 3.23	25.74 ± 3.23	25.90 ± 3.23	0.121
Current smoking (n, %)	2880 (31.7%)	280 (27.5%)	2600 (32.2%)	**0.003**
Family history of CAD (n, %)	1207 (13.3%)	130 (12.8%)	1077 (13.3%)	0.622
Laboratory data				
TC (mmol/L)	4.14 ± 1.12	4.15 ± 1.12	4.14 ± 1.12	0.805
TG (mmol/L)	1.79 ± 1.23	1.81 ± 1.47	1.79 ± 1.20	0.580
LDL-C (mmol/L)	2.51 ± 0.99	2.49 ± 0.92	2.51 ± 1.00	0.532
HDL-C (mmol/L)	1.07 ± 0.29	1.07 ± 0.29	1.07 ± 0.29	0.569
Apo A1 (g/L)	1.34 ± 0.30	1.36 ± 0.30	1.34 ± 0.30	0.086
Apo B (g/L)	0.91 ± 0.28	0.93 ± 0.30	0.91 ± 0.29	0.211
Glucose (mmol/L)	5.85 ± 2.56	5.87 ± 1.80	5.83 ± 1.75	0.551
HbA1c (%)	6.29 ± 1.09	6.47 ± 1.20	6.27 ± 1.09	** < 0.001**
Creatinine (mmol/L)	77.33 ± 17.46	77.93 ± 17.29	77.25 ± 17.48	0.246
Treatment in hospital				
Aspirin (n, %)	8595 (94.5%)	984 (96.8%)	7611 (94.3%)	**0.001**
β-Blockers (n, %)	6846 (75.3%)	802 (78.9%)	6044 (74.9%)	**0.005**
CCBs (n, %)	3356 (36.9%)	403 (39.6%)	2953 (36.6%)	0.057
ACE inhibitors (n, %)	2045 (22.5%)	273 (26.8%)	1772 (21.9%)	** < 0.001**
ARB (n, %)	2150 (23.6%)	291 (28.6%)	1859 (23.0%)	** < 0.001**
Lipid-lowering medication (n, %)	8159 (89.7%)	958 (94.2%)	7201 (89.2%)	** < 0.001**
Anti-diabetes drugs (n, %)	3173 (34.9%)	425 (41.8%)	2748 (34.0%)	** < 0.001**
Coronary revascularization (n, %)	4493 (49.4%)	500 (49.2%)	3993 (49.6%)	0.861
Severity of coronary disease				
NOCAS (n, %)	1290 (14.2%)	90 (8.8%)	1200 (14.9%)	** < 0.001**
ICAS (n, %)	1003 (11.0%)	94 (9.2%)	909 (11.3%)	0.056
SCAS (n, %)	6798 (74.8%)	833 (81.9%)	5965 (73.9%)	** < 0.001**

*MACE* major adverse cardiovascular event, *BMI* body mass index, *LVEF* left ventricular ejection fraction, *CAD* coronary artery disease, *TC* total cholesterol, *TG* triglyceride, *LDL*-*C* low-density lipoprotein cholesterol, *HDL-C* high-density lipoprotein cholesterol, *Apo* apolipoprotein, *HbA1c* hemoglobin A1c, *CCBs* calcium channel blockers, *ACE* angiotensin converting enzyme, *ARB* angiotensin receptor blocker, *NOCAS* non-obstructive coronary artery stenosis, *ICAS* intermediate coronary artery stenosis, *SCAS* severe coronary artery stenosis

Values are expressed as the mean ± SD, or n (%). Bold P means statistically significant

**Fig. 3:**
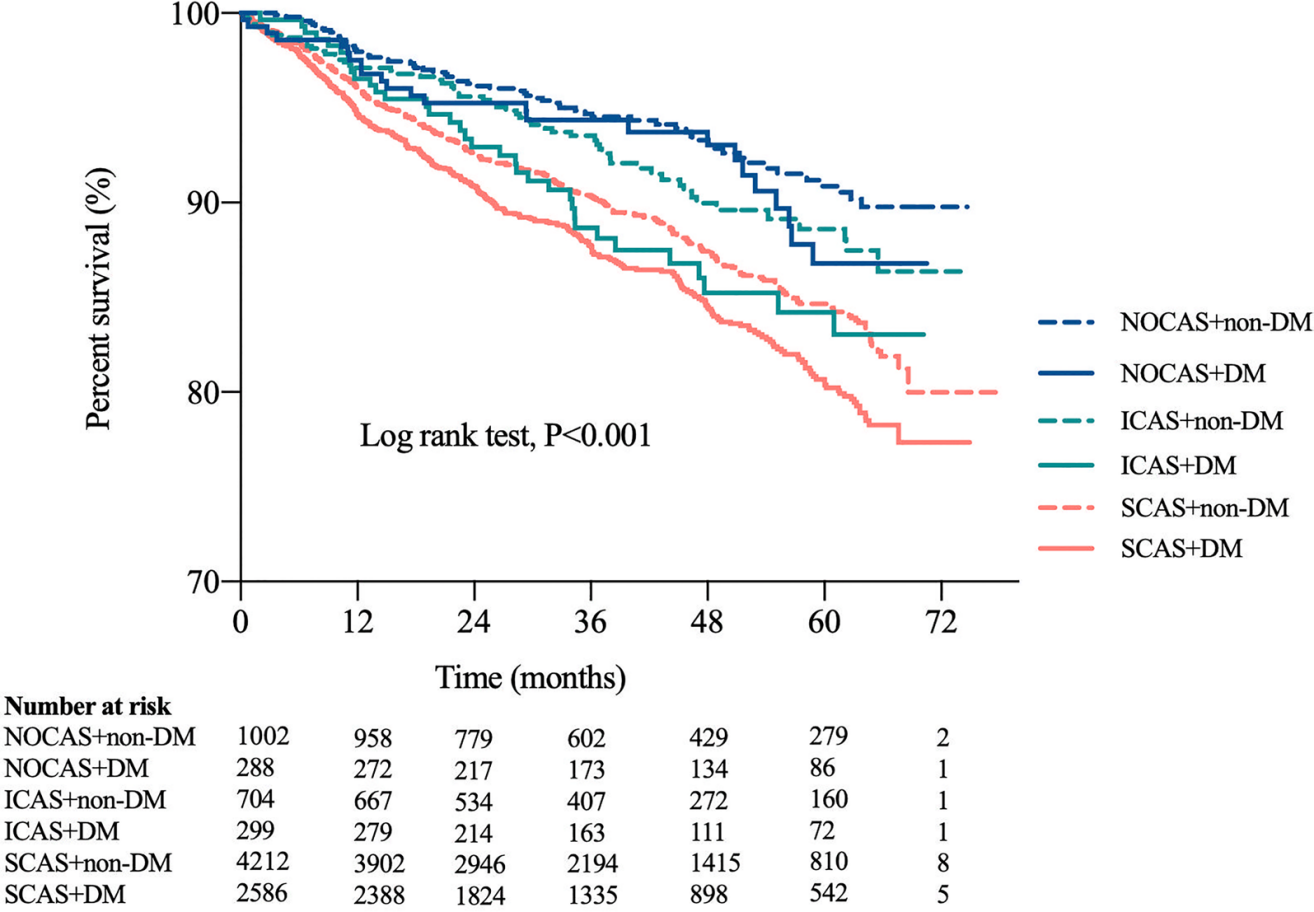
Kaplan–Meier curves in different subgroups. Figure 3 showed the Kaplan–Meier curves in patients with or without diabetes according to the severity of coronary artery stenosis. (NOCAS: non-obstructive coronary artery stenosis; ICAS: intermediate coronary artery stenosis; SCAS: severe coronary artery stenosis; DM: diabetes mellitus)

**Fig. 4:**
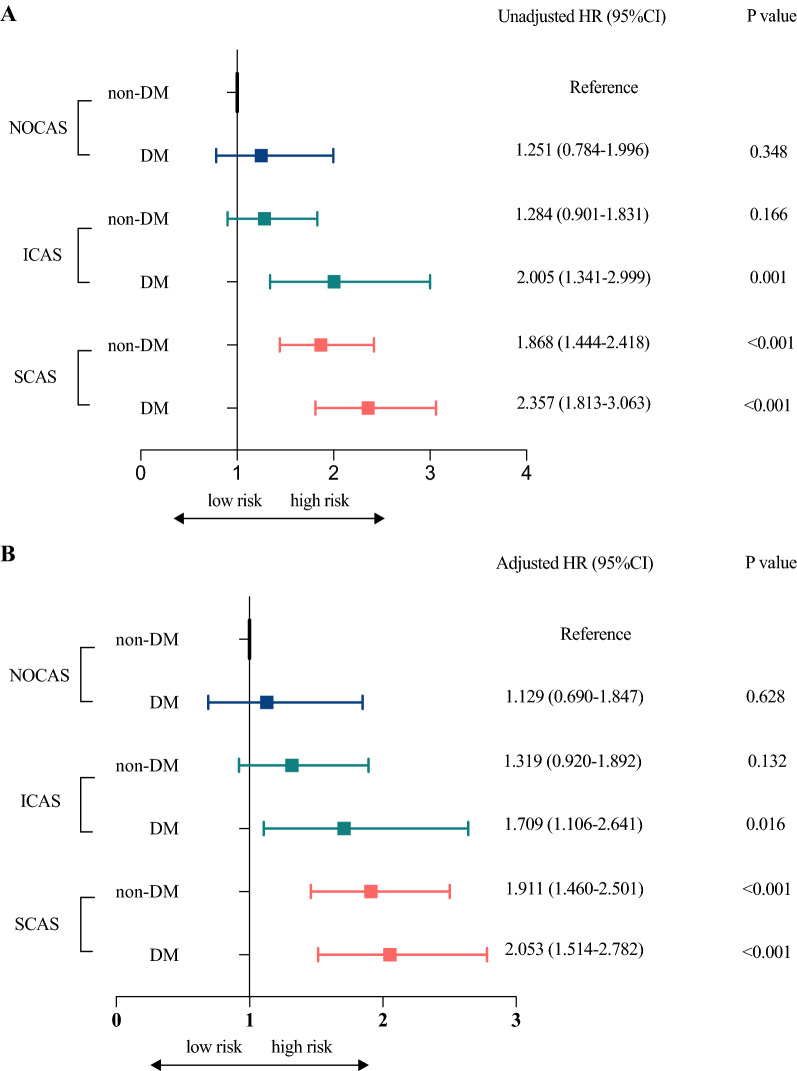
Cox regression analyses of cardiovascular events in different subgroups. **A** showed the unadjusted model. **B** showed the adjusted model after adjustment for age, gender, hypertension, dyslipidemia, body mass index, current smoking, family history of coronary artery disease, glucose, and hemoglobin A1c. (NOCAS: non-obstructive coronary artery stenosis; ICAS: intermediate coronary artery stenosis; SCAS: severe coronary artery stenosis; DM: diabetes mellitus; HRs: hazard ratios; CIs: confidential intervals)

Besides, as shown in Fig. 3, the Kaplan–Meier analysis curves presented the highest risk of MACEs in patients with SCAS and DM compared with other subgroups (log rank test, p < 0.001). Furthermore, we assessed the prognostic utility in patients presented different extent of coronary stenosis with or without DM by Cox regression analysis in Fig. 4. Obviously, in unadjusted model, patients with ICAS and DM, SCAS and non-DM, SCAS and DM showed increased risk of MACEs (Fig. 4A: HR: 2.005; 95% CI 1.341–2.999, p = 0.001; HR: 1.868; 95% CI 1.444–2.418, p < 0.001; HR: 2.357; 95% CI 1.813–3.063, p < 0.001; respectively) compared to those in NOCAS and non-DM group (as reference). Particularly, there was a comparable risk of MACEs in patients with ICAS plus DM and patients with SCAS alone. The results remained after adjusted for age, gender, hypertension, dyslipidemia, BMI, current smoking, family history of CAD, glucose, and HbA1c (Fig. 4B: adjusted HR: 1.709; 95% CI 1.106–2.641, p = 0.016; adjusted HR: 1.911; 95% CI 1.460–2.501, p < 0.001; adjusted HR: 2.053; 95% CI 1.514–2.782, p < 0.001).

## Discussion

In this perspective, observational study on a large Chinese cohort with long-term follow-up, we examined the prognostic association of DM with outcomes in patients with different degree of CAS assessed by CAG. Our data indicated that diabetic patients with ICAS and SCAS were both associated with significantly increased risks of MACEs compared to ones with a mild coronary stenosis, even after adjusting for traditional cardiovascular risk factors. These findings suggested that more precise risk assessment should be performed in diabetic patients with different coronary stenosis.

Previously, a number of observational and prospective studies have reported the relation of coronary stenosis severity to the development of cardiovascular events [24–26]. On the one hand, evidence has revealed that acute coronary events commonly arise from severely stenotic lesions, since patients occurred acute coronary syndrome were found to have severe coronary stenosis [24, 25]. For example, Frøbert et al. have revealed that above 90% of patients with acute MI presented with much than 50% stenosis of coronary arteries, and above 60% of them presented with much than 70% stenosis of coronary arteries [25]. These data suggested that severe lumen stenosis appeared to be an important prerequisite for acute coronary events. On the other hand, patients with severe coronary stenosis were more likely to develop increased cardiovascular events. Data obtained from series of prospective studies supported this notion [19, 26, 27]. For instance, a recent study indicated that physiological stenosis severity assessed by fractional flow reserve was closely related to the plaque vulnerability assessed by CTA, and was significantly associated with the risk of clinical events [27]. Another angiographic study from Zaman et al. also reported that severe coronary stenosis was a valuable predictor for acute coronary events in the subsequent follow-up duration [26]. Results from the international multicenter prospective study, CONFIRM (Coronary CT Angiography Evaluation for Clinical Outcomes: An International Multicenter Registry), were similar to prior studies, which have demonstrated that the extent and severity of CAD was an indicator for poor prognosis [28]. Moreover, they reported that both non-obstructive and obstructive CAD were associated with increased all-cause mortality, whether using a definition of non-obstructive stenosis range from 1 to 49% or 1% to 69% [20]. Based on above studies, we have carefully examined the relation of coronary stenosis to clinical outcomes and tested this interesting phenomenon in Chinese population. In line with the previous studies, our data indicated that patients with severe stenosis (70–100%) of coronary arteries assessed by CAG had the highest clinical events than ones with mild-to-intermediate stenosis (SCAS vs ICAS vs NOCAS: 12.3% vs 9.4% vs 7.0%). Furthermore, we found that patients with ICAS plus DM had a comparable risk of MACEs to patients with SCAS alone (unadjusted HR: 2.005 vs 1.868; adjusted HR: 1.709 vs 1.911).

In fact, it is well-recognized that diabetes is an independent risk factor for the development of CAD, and there was robust evidence demonstrating the high prevalence of adverse cardiovascular risk in diabetic individuals [2–6, 8]. The CONFIRM study indicated that diabetic patients had a higher prevalence and severity of CAD compared with matched patients without DM [8]. Moreover, coronary lesions in patients with DM usually present greater atherosclerotic plaque burden, and are even more complicated characterized by diffuse, calcified, multivessel diseases, and smaller coronary artery lumen diameter than ones without DM [9–11, 29]. A recent study with a small sample of diabetic patients explored the impact of diabetes duration on the extent and severity of coronary stenosis measured by coronary CTA, and they found that longer DM duration was associated with a higher prevalence and severity of CAD as well as risk of MACE, and greater coronary disease had increased risk of MACE independent of co-existing CAD risk factors [7]. However, till now, there is limited information concerning the prognostic association of DM and outcomes in patients with different degree of coronary stenosis. That is the reason why we performed the present study. In our observation, we determined the prognostic performance of coronary stenosis in patients with or without DM for the prediction of MACE. Interestingly, data showed that patients with ICAS plus DM had a comparable risk of MACE to ones with SCAS without DM. This data provided complementary information regarding the association of diabetes, coronary stenosis with outcomes, which expanded previous evidence of the impact of diabetes on clinical outcomes in patients with coronary atherosclerosis.

Given the clinical burden that diabetes exerted on cardiovascular risk complications, the joint evaluations of diabetes and coronary stenosis are of clinical significance for management of these patients. Hence, recent guidelines and expert consensus have been characterized by further emphasizing the assessment risk factors combined with obvious cardiovascular pathological lesions in patients with diabetes [6, 17]. The potential mechanism of diabetes as a risk factor of cardiovascular diseases might because of hyperglycemia influences the pathology of coronary microvascular, inflammation and sympathetic nervous system activity, vasospastic and structural remodeling of circulation, resulting in an increased cardiovascular risk [17, 30]. Although our study did not provide the detailed mechanism exploration, the present study might support the notion that diabetes could impose a bad impact on the adverse outcome in patients with CAD, even in those with intermediate coronary stenosis.

Our study had several limitations. Firstly, as the nature of an observational studies, our study was subject to selection and misclassification bias. Secondly, the coronary stenosis percent was defined by CAG, the evaluation of stenosis was measured by semiquantitative visual analysis rather than by other volumetric quantification and functional tests for the stenotic arteries. Moreover, although the patients enrolled in this study received the optimal medical therapy for secondary prevention when they were discharged from hospital, the detailed data covering medications and hypoglycemic agents were not available during the follow-up. Finally, our study was a single center study, and the sample size in subgroup was relatively small. Therefore, future studies are needed to confirm our findings.

## Conclusions

The present study provided an insight regarding the association of DM with cardiovascular outcomes in patients with different degree of coronary stenosis in a large Chinese cohort. Our results indicated that patients with DM and intermediate stenotic coronary lesion presented worse outcomes and were comparable to patients with severe coronary stenosis alone, suggesting that more intensive managements might be needed in such patients.

## Data Availability

The datasets used and/or analyzed during the current study are available from the corresponding author on reasonable request.

## Abbreviations

CAD: Coronary artery disease
DM: Diabetes mellitus
HbA1c: Hemoglobin A1c
CAG: Coronary angiography
MACE: Major adverse cardiovascular events
HR: Hazard ratio
CI: Confidence interval
